# Diagnostic utility of FDG-PET in the differential diagnosis between different forms of primary progressive aphasia

**DOI:** 10.1007/s00259-018-4034-z

**Published:** 2018-05-09

**Authors:** Femke Bouwman, Stefania Orini, Federica Gandolfo, Daniele Altomare, Cristina Festari, Federica Agosta, Javier Arbizu, Alexander Drzezga, Peter Nestor, Flavio Nobili, Zuzana Walker, Silvia Morbelli, Marina Boccardi, for the EANM-EAN Task Force for the Prescription of FDG-PET for Dementing Neurodegenerative Disorders

**Affiliations:** 1 Department of Neurology & Alzheimer Center, Amsterdam Neuroscience, VU University Medical Center, De Boelelaan 1118, 1081 HZ Amsterdam, The Netherlands; 2 Alzheimer Operative Unit, IRCCS S. Giovanni di Dio, Fatebenefratelli, Brescia, Italy; 3 LANE – Laboratory of Alzheimer’s Neuroimaging & Epidemiology, IRCCS S. Giovanni di Dio, Fatebenefratelli, Brescia, Italy; 4 Department of Molecular and Translational Medicine, University of Brescia, Brescia, Italy; 5 Neuroimaging Research Unit, Institute of Experimental Neurology, Division of Neuroscience, San Raffaele Scientific Institute, Vita-Salute San Raffaele University, Milan, Italy; 6 Department of Nuclear Medicine, Clinica Universidad de Navarra, University of Navarra, Pamplona, Spain; 7 Department of Nuclear Medicine, University Hospital of Cologne, University of Cologne and German Center for Neurodegenerative Diseases (DZNE), Cologne, Germany; 8 German Center for Neurodegenerative Diseases (DZNE), Magdeburg, Germany; 9 Queensland Brain Institute, University of Queensland and at the Mater Hospital Brisbane, Brisbane, Australia; 10 Department of Neuroscience (DINOGMI), University of Genoa, Clinical Neurology and Polyclinic IRCCS San Martino-IST, Genoa, Italy; 11 Division of Psychiatry & Essex Partnership University NHS Foundation Trust, University College London, London, UK; 12 Nuclear Medicine Unit, San Martino Hospital, Department of Health Sciences, University of Genoa, Genoa, Italy; 13 LANVIE (Laboratoire de Neuroimagerie du Vieillissement), Department of Psychiatry, University of Geneva, Geneva, Switzerland

**Keywords:** FDG-PET, Primary progressive aphasia, PPA, Neurodegenerative, Semantic, Logopenic, Agrammatic, Dementia

## Abstract

**Purpose:**

A joint effort of the European Association of Nuclear Medicine (EANM) and the European Academy of Neurology (EAN) aims at clinical guidance for the use of FDG-PET in neurodegenerative diseases. This paper addresses the diagnostic utility of FDG-PET over clinical/neuropsychological assessment in the differentiation of the three forms of primary progressive aphasia (PPA).

**Methods:**

Seven panelists were appointed by the EANM and EAN and a literature search was performed by using harmonized PICO (Population, Intervention, Comparison, Outcome) question keywords. The studies were screened for eligibility, and data extracted to assess their methodological quality. Critical outcomes were accuracy indices in differentiating different PPA clinical forms. Subsequently Delphi rounds were held with the extracted data and quality assessment to reach a consensus based on both literature and expert opinion.

**Results:**

Critical outcomes for this PICO were available in four of the examined papers. The level of formal evidence supporting clinical utility of FDG-PET in differentiating among PPA variants was considered as poor. However, the consensual recommendation was defined on Delphi round I, with six out of seven panelists supporting clinical use.

**Conclusions:**

Quantitative evidence demonstrating utility or lack thereof is still missing. Panelists decided consistently to provide interim support for clinical use based on the fact that a typical atrophy or metabolic pattern is needed for PPA according to the diagnostic criteria, and the synaptic failure detected by FDG-PET is an earlier phenomenon than atrophy. Also, a normal FDG-PET points to a non-neurodegenerative cause.

## Introduction

In the lack of clinical guidelines for the use of FDG-PET to diagnose dementing neurodegenerative conditions, the European Association of Nuclear Medicine (EANM) and the European Academy of Neurology (EAN) launched a joint effort aimed at providing clinicians with clinical guidance for using the exam. To this avail, a set of 21 clinical questions was defined to perform literature searches and assessment of the evidence supporting FDG-PET clinical use, and feeding a group of experts defining consensual [1].

In this paper, we report the availability of evidence supporting the use of FDG-PET for the differential diagnosis between different forms of primary progressive aphasia (PPA). PPA is divided into clinical variants based on specific speech and language features characteristic of each subtype. Clinical criteria for the three variants of PPA—nonfluent/agrammatic, semantic, and logopenic—were developed by an international group of PPA investigators [2]. This classification can be further specified as “imaging-supported” if the expected pattern of atrophy or hypometabolism is found, and “with definite pathology” if pathologic or genetic data are available. Although FDG-PET is part of the classification described by Gorno-Tempini and colleagues’, criteria [2] were not based on quantitative literature analysis. Nevertheless, FDG-PET is used on a regular basis in clinical practice for the diagnosis of PPA types.

In this study, we thus assessed the quality of available evidence supporting the utility of FDG-PET in the differential diagnosis of PPA variants, which is relevant for indicating a diagnosis and prognosis in individual patients.

## Methods

EANM and EAN appointed respectively four and three panelists to produce recommendations based on the incremental value of FDG-PET, as added on clinical-neuropsychological examination, to differentiate among clinical presentations (non-fluent/agrammatic form, semantic dementia, logopenic aphasia). Consensus recommendations were developed through a Delphi procedure [1], where panelists voted based on the information about the availability and quality of evidence, assessed by an independent methodological group [3], and on their own expertise.

Briefly, we performed literature searches using harmonized PICO (Population, Intervention, Comparison, Outcome) question keywords edited by panelists; we screened the studies for eligibility, extracted the data to assessed their methodological quality, and provided an evidence assessment consistent with the EFNS guidance [4] in the specific context of FDG-PET studies (this issue) [3].

### PICO question

For this paper, the PICO question was whether *FDG-PET should be performed, as adding diagnostic value (in terms of accuracy, and* versus *pathology or biomarkers of brain amyloidosis), as compared to standard clinical/neuropsychological assessment alone, to differentiate among clinical presentations (non-fluent/agrammatic form, semantic dementia, logopenic aphasia) and to obtain indirect information on the molecular pathologies in patients with primary aphasias*. Note that, within the whole initiative, the incremental diagnostic value of FDG-PET versus clinical diagnosis was meant in terms of increased accuracy of the nosological diagnosis. Other exams, and particularly amyloid PET or CSF biomarkers in this case, were expressively not included in the PICO question, nor in the Delphi sessions, not being the focus of the project.

### Eligibility criteria

Only original full papers published in English on international impacted journals were considered, excluding reviews, management guidelines, abstracts, and gray literature. Any sample size was allowed if pathology was the gold standard for diagnosis. Otherwise, the minimum sample sizes for including papers was five.

### Literature search

Electronic search strategy, developed and tested with panelists, was performed through predefined strings, specific to the PICO question, and including a selection of terms taken from a largely inclusive literature selection, in order to pick all variants for the same keyword [3].

Literature searches were performed using the Medline and Embase databases, and included literature published by April 2016. In reporting the findings of this review, we adhered to the standards of the Preferred Reporting Items for Systematic Reviews and Meta-Analyses (PRISMA) [5]. A first independent screening of all included studies was performed by a neurologist, who could include additional papers based on personal knowledge or tracking from references of papers. The full texts of potentially eligible studies were then independently reassessed for eligibility by the methodological team.

### Data extraction and quality assessment

We extracted data to evaluate the quality of evidence in support to the clinical use of FDG-PET for PPA as described by Boccardi and colleagues [3]. Data extractors for this review were SO and FG. The quality of evidence was assessed consensually within the methodological group based on study design, gold/reference standard, FDG-PET image assessment (visual or semi-quantitative methods), risk of bias, index test imprecision, applicability, effect size, and effect inconsistency. Critical outcomes were validated measures of test performance (accuracy, sensitivity, specificity, AUC, positive and negative predictive values, and likelihood ratios). A final assessment of *relative availability of evidence* was formulated, keeping into account the evidence availability among all of the 21 PICOs. This ranking was summarized as very poor/lacking, poor, fair, or good.

## Results

**Fig. 1:**
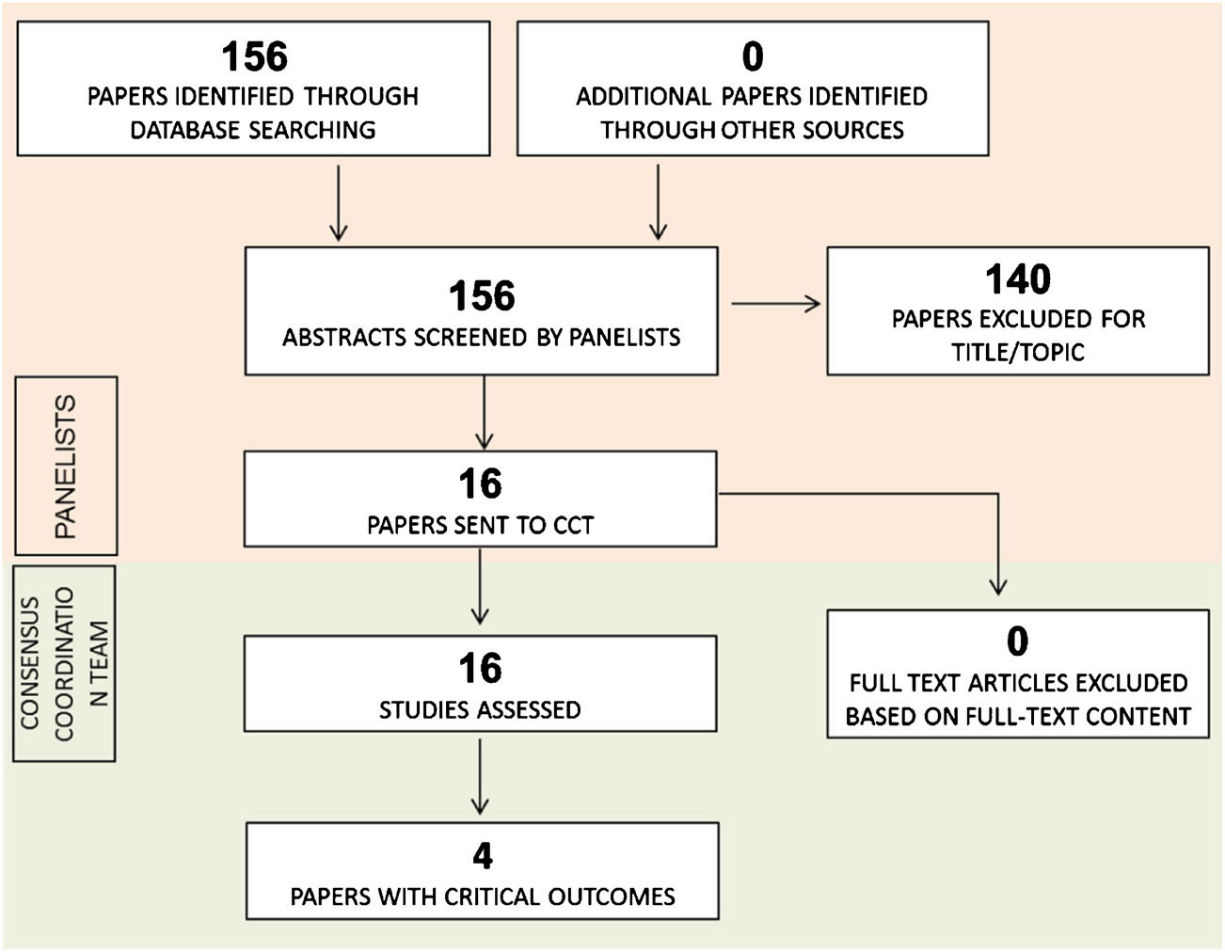
PRISMA flowchart of selected papers for PICO 16 regarding requirement of semi-automated assessment (adapted from Moher et al. 2009) [5]

Among the 156 papers identified and screened by the referent panelist (FB), 16 reported the comparison of interest (Fig. 1) and were included in the assessment. Critical outcomes for this PICO were available in four of the examined papers [6–9] (see Tables 1 and 2 PICO 16; data extraction table available at (https://drive.google.com/drive/u/0/folders/0B0_JB3wzTvbpVFYtUGxHdGZWYmc).

Using SPM for the assessment of FDG-PET, Matias-Guiu and colleagues [7] found that sensitivity, specificity, and accuracy in differentiating all PPA variants were 86.2, 66.7, and 84%, respectively, using clinical diagnosis as reference diagnosis. In addition, they reported sensitivity, specificity, and accuracy of SPM in differentiating among the three variants of PPA. In detail, sensitivity was of 91.6% for av-PPA, 100% for sv-PPA, and 78.6% for lv-PPA, specificity was 100% for av-PPA, 93.1% for sv-PPA and 94.4% for lv-PPA and accuracy was 97% for av-PPA, 94% for sv-PPA, and 87% for lv-PPA, respectively. They also reported a positive predictive value of 96.1% (100% for av-PPA, of 60% for sv-PPA, and 91.7% for lv-PPA, respectively) and a negative predictive value of 33.3% (95.2% for av-PPA, 100% for sv-PPA, and 85% for lv-PPA) (Table 1).

**Table 1:** PICO 16 (PART 1). The quality of evidence for each critical outcome in differentiating among clinical presentations in PPA patients. The overall quality of evidence was assessed as described in section 2.4, and ranked among the 21 PICOs of the whole project (Nobili, Arbizu et al. 2018) to provide information about availability of evidence relative to the FDG-PET field (see [3] for further details)

PICO 16: Differentiate among clinical presentations in PPA patients											
Critical outcomes	No. of papers	Sample size	Gold/reference standard	Risk of bias	Index test imprecision	Applicability	FDG-PET assessment	Effect range (CI)	Effect assessment	Effect inconsistency	Outcome quality
Sensitivity	1	12 av-PPA 3 sv-PPA 14 lv-PPA	Clinical diagnosis	Not serious	Serious	Serious	Semi-quantitative (SPM)	- av-PPA: 91.6% (CI: 61–100%). - sv-PPA: 100% (CI: 29–100%). - lv-PPA: 78.6% (CI: 49–95%). - All variants: 86.2% (CI: 68–96%)	HI	NA	LOW
Specificity	1	12 av-PPA 3 sv-PPA 14 lv-PPA	Clinical diagnosis	Not serious	Serious	Serious	Semi-quantitative (SPM)	- av-PPA: 100% (CI: 83–100%). - sv-PPA: 93.1% (CI: 77–99%). - lv-PPA: 94.4% (CI: 73–100%). - All variants: 66.7% (CI: 9–99%)	HI	NA	LOW
Accuracy	1	12 av-PPA 3 sv-PPA 14 lv-PPA	Clinical diagnosis	Not serious	Serious	Serious	Semi-quantitative (SPM)	- av-PPA: 97% (CI: 84–100%). - sv-PPA: 94% (CI: 79–99%). - lv-PPA: 87% (CI: 71–96%). - All variants: 84% (CI: 67–95%)	HI	NA	LOW
PPV	1	12 av-PPA 3 sv-PPA 14 lvPPA	Clinical diagnosis	Not serious	Not serious	Serious	Semi-quantitative (SPM)	- av-PPA: 100% (CI: 71–100%). - sv-PPA: 60% (CI: 15–95%). - lv-PPA: 91.7% (CI: 61–100%). - All variants: 96.1% (IC: 80–100%)	HI	NA	LOW
NPV	1	12 av-PPA 3 sv-PPA 14 lv-PPA	Clinical diagnosis	Not serious	Not serious	Serious	Semi-quantitative (SPM)	- av-PPA: 95.2% (CI: 76–100%). - sv-PPA: 100% (CI: 87–100%). - lv-PPA: 85% (CI: 62–97%). - All variants: 33.3% (CI: 4–78%)	HI	NA	LOW
**RELATIVE AVAILABILITY OF EVIDENCE: POOR**											

In their study, Nestor and colleagues [6] analyzed sensitivity and specificity of FDG-PET in predicting AD pathology in five patients with autopsy confirmation. They found that bilateral hypometabolism in the temporo-parietal cortex had 50% sensitivity in detecting AD pathology, while normal temporo-parietal cortex had 38% sensitivity in detecting non-AD pathology; bilateral hypometabolism in the temporo-parietal cortex had 100% specificity in detecting AD pathology and normal temporo-parietal cortex had 100% specificity in detecting non-AD pathology. Notably, they also found that a unilateral left temporoparietal lesion did not discriminate AD from FTLD, which is the reason for the low sensitivities in spite of high specificity [6].

According to Whitwell and colleagues [8], using SPM and ROI analyses, right lateral temporal hypometabolism, and asymmetric hippocampal metabolism had 67 and 83% sensitivity, respectively, and 100% specificity in predicting amyloid-negative lv-PPA. Finally, in Taswell and colleagues [9], using 3D SSP analyses, the PPV value in predicting AD pathology was > 90% both in the lv-PPA and av-PPA, while NPV was greater in av-PPA (96%) and sv-PPA (92%) than in lv-PPA (81%). Both of these papers used amyloid PET for confirming the underlying pathology.

The assessed studies caused concerns regarding risk of bias for patient selection and the applicability of the index test, being semi-quantitative methods for image analysis still uncommon in clinical centers. The large heterogeneity of comparisons, besides the very few studies and patient number, does not allow to support consistency of results (Table 1 and 2).

**Table 2:** PICO 16 (PART 2). Table reports the quality of evidence for each critical outcome in detecting the underlying molecular pathologies (e.g., amyloidosis or tauopathies) in PPA patients

	PICO 16: Detecting the underlying molecular pathologies (e.g., amyloidosis or tauopathies) in PPA patients										
Critical outcomes	No. of papers	Sample size	Gold/reference standard	FDG-PET assessment	Risk of bias	Index test imprecision	Applicability	Effect range (CI)	Effect assessment	Effect inconsistency	Outcome quality
Detect AD pathology	2	53 PPA	1 Pathology 1 Biomarker-based diagnosis	1 Unclear 1 Semi-quantitative	Serious	Very serious	Very serious	Study 1. - Accuracy: 91% (Standard error: 7) in lv-PPA, 94% (6) in av-PPA, 85% (10) sv-PPA. - PPV: 94% in lv-PPA, 92% in av-PPA, NA in sv-PPA. - NPV: 81% in lv-PPA, 96% in av-PPA, 92% in sv-PPA. - LR+: 1.91 in lv-PPA, 11.0 in av-PPA, NA in sv-PPA. - LR-: 0.09 in lv-PPA, 0.10 in av-PPA, 1.10 in sv-PPA. Study 2. - Sensitivity: 50% (CI NA). - Specificity: 100% (CI NA)	Moderate	NA	LOW
Detect non-AD pathology	2	31 PPA	1 Pathology 1 Biomarker-based diagnosis	1 Unclear 1 Semi-quantitative	Serious	Very serious	Very serious	Study 1. - Sensitivity: 83% (CI: 36–100%). - Specificity: 100% (CI: 83–100%). - AUC: 90% (CI NA). - PPV: 100% (CI: 48–100%). Study 2. - Sensitivity: 38% (CI NA). - Specificity: 100% (CI NA)	Moderate	NA	LOW
**RELATIVE AVAILABILITY OF EVIDENCE: POOR**											

Risk of bias: assessment of the study design and other methodological features (e.g., patient selection, clinical diagnostic criteria used)

Index test methods: assessment of index test methodology (e.g., technical details, image analysis methods and statistical analysis)

Applicability: representativeness of the studied population and index test reproducibility in clinical practice (semi-quantitative methods correspond to ‘serious’ indirectness, visual + semi-quantitative methods correspond to ‘not serious’ indirectness, due to partial implementation of quantitation in clinical practice)

Effect: lowest and highest values for each critical outcome; when more values were obtained for the same outcome, the highest was reported

Effect assessment: 51–70% low, 71–80% moderate, 81–100% high

Effect inconsistency: ‘Not serious’ if lowest and highest values difference was 0–20, ‘serious’ 21–40, ‘very serious’ > 40

Outcome quality: summary of evidence as from all columns

Taking into account the availability of formal evidence for all of the PICOs within the entire project, the level of evidence supporting clinical utility of FDG-PET in differentiating among PPA variants was considered as poor. The consensual recommendation was defined on Delphi round I, with six out of seven panelists supporting clinical use. Panelists kept that specific patterns of atrophy and/or hypometabolism are necessary for the diagnosis of PPA according to the diagnostic criteria and FDG-PET is more sensitive than MRI [2].

## Discussion

In this paper, we assessed the evidence on the clinical utility of FDG-PET for the differential diagnosis between different forms of PPA as an add on to clinical diagnosis, and without comparison with additional exams. We show that literature evidence for the use of FDG-PET in PPA is poor. Nevertheless, six out of seven panelists, clinically experienced in diagnosing PPA, supported clinical use of FDG-PET in the first Delphi round, for differentiation between PPA types.

During the Delphi round, panelists indicated that clinically it may be challenging to differentiate the three types of PPA. Actually, since the publication of the diagnostic criteria by Gorno-Tempini [2], many researchers showed overlap between the different PPA variants both clinically and on imaging [11–13]. Still the PPA diagnostic criteria of Gorno-Tempini clearly indicate that the typical atrophy or metabolic pattern of the three main forms are mandatory for the diagnosis. In this context, the synaptic failure detected by FDG-PET is an earlier phenomenon than atrophy and thus this tool was still indicated by the panelists as extremely useful (Table 3). In addition, the panelists remarked that a normal FDG-PET scan may also have diagnostic value, as it points to a non-neurodegenerative cause of clinical aphasic symptomatology.

**Table 3:** Availability of evidence and panelists’ decisions supporting the use of FDG-PET in the differential diagnosis of PPA variants

PICO	Relative availability of evidence	Panelists’ recommendations	Main reasons for final decision
16 – diagnosis of PPA	Poor	Yes	More sensitive than MRI. Required in PPA diagnostic criteria

In their diagnostic criteria, Gorno-Tempini et al. make use of MRI, FDG-PET, and SPECT as imaging modalities. Since MRI is mandatory in all patients also for the exclusion of other non-neurodegenerative underlying etiologies, some of the patients might show (on MRI) a pattern of atrophy already clearly suggestive for a subtype of PPA. A systematic investigation of the potential added value of FDG-PET in patients with pattern of atrophy already supporting the diagnosis of PPA is outside the aim of the present study and should be addressed when discussing a complete diagnostic algorithm for PPA. With respect to perfusion SPECT, due to the worse resolution, SPECT should not be performed when FDG-PET is available [14].

Considering the recent advances in molecular imaging and currently available neuropathological biomarkers for Alzheimer’s disease (AD), i.e., CSF abeta, tau, ptau, and amyloid PET, the focus of this paper covers only a part of the comparative analyses that should be performed to outline a complete and cost-effective diagnostic algorithm. Indeed, the added value of FDG-PET over both clinical/neuropsychological evaluation and specific AD biomarkers needs to be addressed. Especially since a negative amyloid marker firmly rules out AD, although co-pathology of AD may occur in sv-PPA and av-PPA, yielding positive amyloid biomarkers, while the causative underlying pathology may be non-AD (e.g., TDP43 or tau pathology). The hypometabolic pattern on FDG-PET is supportive in these cases providing information on the extent and localization of neuronal dysfunction, and thus the endophenotype of neuronal injury. In this way, FDG-PET may give indirect information on a supposedly underlying molecular pathology, such as TDP43 pathology in semantic dementia. Actual and direct information on the underlying neuropathology naturally requires more specific biomarker and/or genetic analysis. All in all, though, the aim of the EANM-EAN initiative, and of this paper, was limited to assessing the incremental value of FDG-PET over clinical assessment, to give a guideline specific to the use of FDG-PET in a clinical setting. We should also consider that both CSF analysis as well as amyloid PET are not widely available in all memory clinics yet, whereas FDG-PET is a widely used technique also in other specialties such as oncology, and therefore widely available in most memory clinics. In the future, when CSF analysis, amyloid PET, and possibly other neurodegenerative biomarkers and/or tracers (i.e., amyloid and/or tau) become widely available for clinical purposes, a formal comparison with FDG-PET will be needed to define a cost-effective algorithm for diagnosis in PPA and dementia in general [15, 16].

Panelists also mentioned that solving standardization issues (e.g., for scan reading or normality threshold) may overcome some of the current heterogeneity in FDG-PET diagnostic performance [16, 17], as did also the other literature reviews within the EANM-EAN Initiative [18–23].

The main limitation of the present study consisted in the fact that the evidence assessment had to be performed, to the best of our methodological resources, on literature characterized by important methodological limitations. In order to evaluate and compare quantitatively the incremental value of diagnostic tests, only papers reporting validated measures of test performance could be included for analysis. Thereby, as many as 12 out of the 16 papers found in the literature had to be excluded, although they performed investigations potentially relevant to the PICO question. Information on sensitivity and specificity, and, even more, measures of test performance that be independent on the prevalence of the disease in the population (i.e., PPV, NPV, or negative and positive likelihood ratios) are mandatory to allow assessment of incremental diagnostic value, and thus the definition of evidence-based diagnostic guidelines. To allow comparison of diagnostic methods such as FDG-PET, we encourage research groups to compute and report these critical outcome measures in future publications, since this can usually be easily done with the data normally collected in this kind of study.

On the other hand, the lack of direct quantification of patient outcomes (health, quality of life, mortality, institutionalization) following FDG-PET-based diagnosis is a main limitation not solvable in the short term. However, even accepting accuracy studies as proxies for more appropriate patient management [24, 25], many limitations remain. Indeed, demonstration of diagnostic improvement after FDG-PET is limited by the lack of pathology confirmation and of head-to-head comparison between FDG-PET and clinical assessment versus the same gold standard [3]. In addition, the frequent use of mere baseline clinical diagnosis as the reference standard conveys the limitation of the intrinsic circularity between hypometabolic patterns and clinical syndromes, and prevents computation of test performance independent of the actual prevalence of the disorder in the examined population. This may be due to the absence of stakeholders, specific to FDG-PET and the so-called ‘orphan’ drugs: no company ever having exclusivity on the radiopharmaceutical, rigorous expensive studies are scarce.

Also for this set of reasons, these recommendations are defined late compared to other diagnostic appropriate use criteria, e.g., those for amyloid-PET [26], which are also based on expert consensus, being defined in the complete lack of data on clinical utility. With frequent gaps in formal evidence, the use of clinicians’ experience should at present be seen as interim evidence.

## Conclusions

Notwithstanding the heterogeneity of results and poor evidence in the literature, consensus was achieved on Delphi Round I, when six out of seven panelists (being experienced clinicians in neurodegeneration of both neurology (EAN) and nuclear medicine (EANM)) supported clinical use of FDG PET in PPA. With synaptic failure being an earlier phenomenon than atrophy, FDG-PET is particularly valuable for the differential diagnosis between different forms of primary progressive aphasia. This recommendation may be weighted based on the availability of other kinds of examination more directly investigating underlying pathophysiology, which comparison was not the focus of the present paper.
